# The future of neurosurgical oncology lies in cognitive neurosciences: understanding brain processing is much more valuable than surgical technology

**DOI:** 10.3389/fonc.2026.1873116

**Published:** 2026-06-11

**Authors:** Hugues Duffau

**Affiliations:** 1 Department of Neurosurgery, Gui de Chauliac Hospital, Montpellier University Medical Center, Montpellier, France; 2 Team “Plasticity of Central Nervous System, Stem Cells and Glial Tumors,” National Institute for Health and Medical Research (INSERM), U1191 Laboratory, Institute of Functional Genomics, University of Montpellier, Montpellier, France

**Keywords:** awake neurosurgery, brain mapping, connectome, neuro-oncology, neuroplasticity, neuroscience

## Introduction

Since many decades, glioma surgery was conceived as a “dilemma”, given that the main priority for (surgical) neurooncologists was to increase the overall survival (OS) of brain tumor patients (1) - but possibly at the expense of quality of life (QoL). Indeed, tumorological considerations received predominant attention, especially by studying OS as the first endpoint in glioblastomas (due to short life expectancy) while by using surrogates in low-grade gliomas (LGG) because a long time is needed before to reach OS. Thus, two major parameters were regularly evaluated, namely, progression-free survival (PFS) and time to next intervention (TNI), since both seemed to be correlated to OS. However, such surrogates were not systematically predictive of survival, even in randomized controlled trials (RCT). Typically, in the EORTC22845 trial which compared early versus delayed radiation therapy (RT) in LGG patients, whereas PFS was significantly longer after early RT, OS was similar in both groups (2), i.e., with no actual benefit from the patient’s perspective. A recent series even showed a markedly diminished OS after early RT compared with deferred RT (despite similar TNI) (3). Yet, the same mistakes were repeated over years. For example, the recent RCT about IDH-inhibitors in LGG reported an impact of Vorasidenib on PFS and TNI with only 14.2 months of median follow-up (4). Thus, no reliable conclusions can be drawn concerning the long-lasting role of this therapy in LGG patients currently living more than 15 years after upfront maximal surgical resection (5–7).

Here, the purpose is to suggest new directions to solve the onco-functional dilemma in surgical neurooncology, based on a paradigmatic shift lying on the integration of cognitive neurosciences as a key factor in the management of glioma patients, to optimize *both QoL and OS*.

## The traditional dogma of tumor-guided resection

In the classical oncological view, the ultimate goal was to remove a brain tumor as extensively as possible. Consequently, surgical technological advances have mostly been oriented toward a better visualization of the supposed “tumor mass”, which in essence does not exist in diffuse gliomas (whatever the grade) due to the migration of tumoral cells within the parenchyma, mainly along the white matter tracts (8). Historically, neuronavigation was erected as a critical intraoperative tool to identify the glioma “borders” despite the brain shift, even though a RCT did not find any significant difference by comparing outcomes with versus without the use of this device (9). Similarly, intraoperative MRI was proposed to guide glioma removal: if a RCT evidenced an improvement of the extent of resection (EOR) thanks to this technology, the rate of neurological complications was high (13%) (10). Further techniques such as fluorescence and Raman spectroscopy were proposed to “see” the neoplasm on-line, but they do not give any functional information about the brain (11, 12). To limit neurological morbidity, functional neuroimaging (fMRI) and diffusion tensor imaging (DTI) tractography were then introduced for preoperative planning and potentially incorporated in a neuronavigational system. However, their limitations and pitfalls should be reminded. Indeed, fMRI cannot distinguish cortical areas critical for brain functions (which must be surgically preserved) from those which can be functionally compensated (thus which can be excised) (13). Regarding DTI, beyond the lack of reproducibility underlined by the experts who showed various tracts reconstructions from the same dataset according to the protocol used (14), tractography does not provide any direct information about the actual function of the white matter bundles (15).

In synthesis, after several decades of technological race with encouraging results regarding EOR increase (eventually with a decrease of immediate postoperative severe morbidity as a secondary endpoint), the key message is that a significant improvement of *both OS with long-term preservation of QoL* was not clearly demonstrated. This is particularly true in LGG patients, in whom the use of an alternative functional-based surgical philosophy resulted in a prolonged OS>20 years while maintaining an active life for >14–15 years (6, 7).

## A shift to “meta-network guided surgery”: the need to integrate cognitive neurosciences to develop a functional connectome-based neuro-oncology

In image-guided surgery that prioritized tumor exploration, the brain was neglected. A rigid view of cerebral processing prevailed, resulting in serious limitations in the management of glioma patients. First, it is still considered that gliomas involving “eloquent” areas (according to the localizationist dogma) cannot be removed, as supported by the fact that hundreds of patients operated on by the author came from other institutes in which they were not selected for surgery because the neoplasm was located within “Broca’s area” or “Wernicke’s area” or “Rolandic area” (6, 16). Second, the myth of “right non-dominant” hemisphere led to the extreme reverse, i.e., to perform surgery for right tumors without any mapping, as this hemisphere was not critical for cognition, emotion and behavior: however, a recent meta-analysis evidenced that resection of right-sided high-grade and low-grade gliomas under awake mapping was a safer procedure with a better preservation of high-order cognitive functions and a higher rate of return to work (RTW) than resection under general anesthesia, despite similar EOR (17). Indeed, neurosurgeons want to avoid hemiplegia and aphasia, but they are not (yet) sensitized to the importance of preventing deteriorations of such higher-order functions, which could result in more subtle impairments not visible to standard neurological examination but with considerable negative consequences on daily familial and socio-professional activities – usually not evaluated (18). Third, the dynamics of neural networks is not taken into consideration, since the surgical planning is based on static preoperative imaging, such as fMRI/DTI (despite their drawbacks), secondarily incorporated in neuronavigation in the operating room (OR), as if the brain was not reorganized in the meantime. Of course, if the aim is to avoid hemiparesis, it can be claimed that the pyramidal pathway is still in the same position and that this information is good enough. Nonetheless, if the purpose is to give all chances for patients to resume an active life, including RTW, preservation of behavioral functions relying on interactions between circuits underpinning complex movement, language, executive functions, memory, mentalizing and so on is impossible by using simplistic information provided by surgical technologies (18). Similarly, neurosurgeons do not integrate the crosstalk across glioma progression (kinetics, pattern of diffusion along the connectivity…) and neural circuits reconfiguration to develop multistage surgical approach, i.e., by removing only a part of the diffuse neoplasm during a first resection, then facilitating brain reallocation thanks to postoperative cognitive rehabilitation and RTW, and to reoperate a few months/years later by increasing the EOR while preserving higher-order functions (19). Such a strategy is made difficult by current guidelines which recommend the administration of adjuvant treatments after incomplete resection, especially RT (20) - despite the risk of limiting the neuroplastic potential and of generating cognitive and emotional disturbances (21, 22). In summary, even though it is now common to speak about the “connectome” in neurooncology (based upon static DTI wrongly considered as providing “functional” information), such terminology here does not reflect the complexity of the interactions within and between neural system.

In parallel, another surgical ideology emerged, relying on the real-time cortico-axonal stimulation mapping and monitoring of brain functioning in awake patients (23). Such an original concept emphasizes the perpetual changes in the neural circuitry, allowing adapted human behavior and opening the door to a considerable potential of neuroplasticity (24), especially for optimizing EOR (even in “eloquent” areas) while maintaining a high level of QoL. This new principle of “meta-networking” (network of networks) guided surgery enabled a dramatic change in the surgical practice, in the long-lasting onco-functional outcomes, and in the development of a new field of “behavioral surgical neurooncology” by bridging the gap between neurosurgery and basic neurosciences (25).

## Discussion: current results and future perspectives

By applying this surgical strategy to glioma resection, namely by deciphering cerebral dynamics at the individual level (“paradoxically” without the use of technology in the OR, especially no neuronavigation, no intraoperative MRI, no fMRI/DTI, no microscope, no robot, no artificial intelligence, etc…) (23), optimized onco-functional outcomes were obtained, in agreement with the two main wishes of patients – namely, to live longer and better (beyond surrogates as PFS or TNI). A better understanding of neural processing in awake patients permitted to solve the old dilemma in neurooncology by reversing the deep purpose, i.e., to pursue the resection up to the cortico-subcortical pathways critical for brain functions, without trying to detect in priority the neoplastic disease in the OR – even though eventually enabling to achieve a supratotal resection when functional networks were distant from the tumor visible on the imaging (representing in fact only the tip of the iceberg) (6, 25).

**Figure 1:**
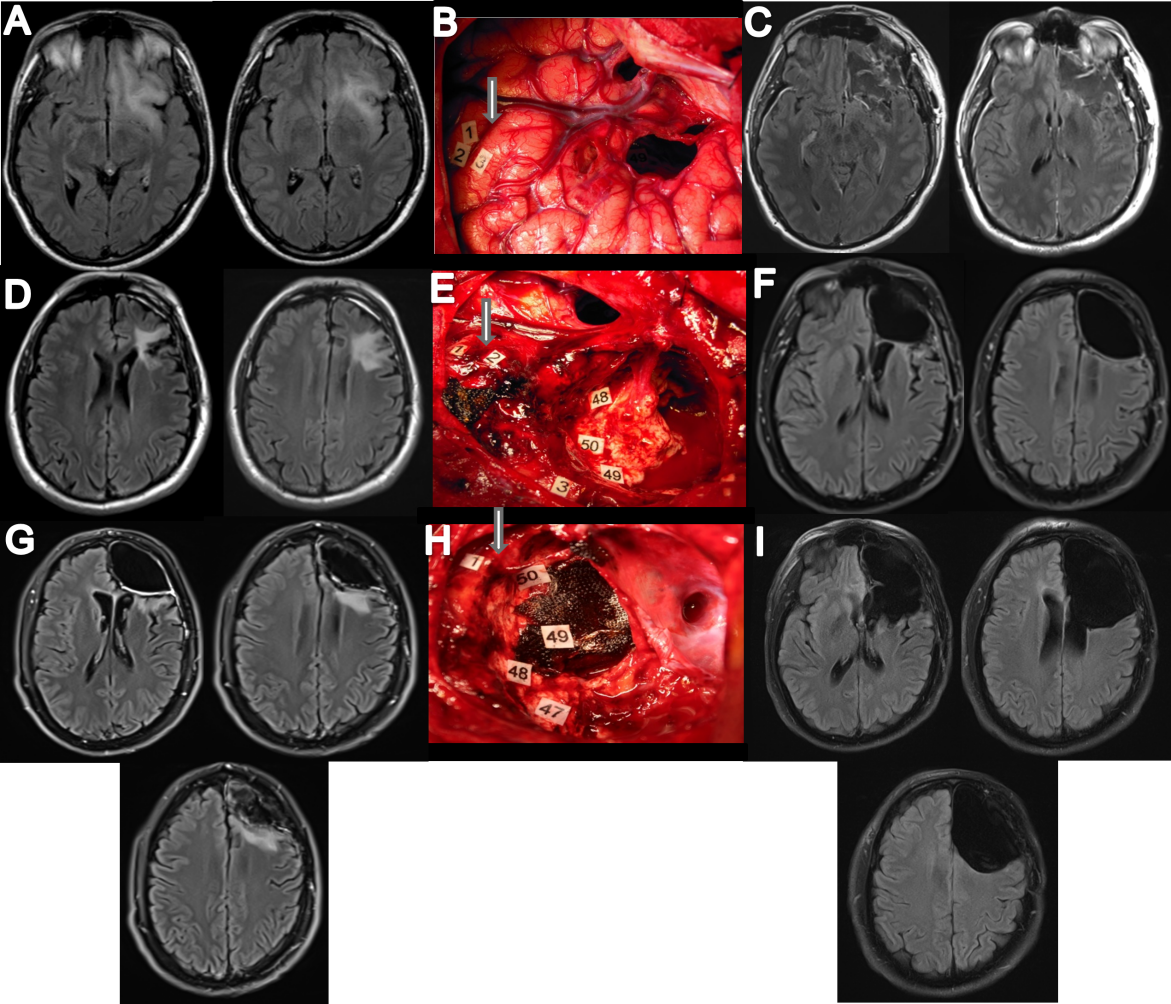
Iterative awake connectome-based resections of a left fronto-insular LGG. **(A)** Preoperative axial FLAIR-weighted MRI achieved in January 2014 in a 36-year-old man, lumberjack, ambidextrous, who experienced a mild brain injury that resulted in the incidental discovery of a left fronto-insular LGG with a volume about 20 mL. The neurological examination was normal. Awake surgery was proposed. **(B)** Intraoperative view after resection in awake patient. The anterior part of the left hemisphere is on the right and its posterior part is on the left. Connectome-based resection has been achieved up to eloquent structures, both at cortical and subcortical levels, especially with respect to the motor, language (counting, naming) and cognitive (multitasking) aspects (number tags show zones of positive direct electrical stimulation). Arrow = precentral sulcus. **(C)** Postoperative axial FLAIR-weighted MRI achieved 3 months after resection, demonstrating a complete resection of the FLAIR signal abnormalities. The patient resumed a normal family, social and professional life within 3 months following surgery, with no seizures. The neuropathological examination revealed a WHO grade II codeleted oligodendroglioma. No adjuvant medical treatment was given: a regular surveillance was decided, with MRI every 6 months. **(D)** Control axial FLAIR-weighted MRI performed in August 2018, in a patient still asymptomatic, evidencing a slow but objective regrowth of the FLAIR hypersignal around the surgical cavity, with no enhancement. Therefore, a reoperation was proposed 4 years after the first resection. **(E)** Intraoperative view after resection in awake patient. The anterior part of the left hemisphere is on the right and its posterior part is on the left. As during the first surgery, a connectome-based resection has been performed, with maximal removal according to cortical and subcortical networks critical for brain functions, especially with respect to the motor, language, and cognitive (semantics, multitasking) aspects (number tags show zones of positive direct electrical stimulation). Interestingly, the resection was increased posteriorly in comparison with the first surgery, thanks to changes in the functional map. Arrow: precentral sulcus. **(F)** Postoperative axial FLAIR-weighted MRI performed 3 months after the second operation, demonstrating a radical resection. The patient recovered quickly and returned to a normal life within the 3 months following reoperation, without any seizures The pathological examination confirmed a WHO grade II oligodendroglioma, with no sign of malignant transformation. No oncological medical treatment was administrated. **(G)** Control axial FLAIR-weighted MRI performed in March 2022, in a patient still asymptomatic, showing a new relapse of the FLAIR hypersignal in the posterior part of the surgical cavity, with no enhancement. Again, a reoperation was proposed 8 years after the first resection. **(H)** Intraoperative view after resection in awake patient. The anterior part of the left hemisphere is on the right and its posterior part is on the left. A functional-guided resection has been achieved, with maximal removal according to cortical and subcortical networks critical for brain functions, especially with respect to the motor, language, and cognitive (semantics, multitasking) aspects (number tags show zones of positive direct electrical stimulation). Remarkably, the resection was increased posteriorly in comparison with the two previous surgeries, thanks to remapping which occurred in the meantime – allowing this time to push this resection in the contact of the precentral sulcus. Arrow: precentral sulcus. **(I)** Postoperative axial FLAIR-weighted MRI achieved 3 months after the third surgery, demonstrating a total resection. The patient recovered quickly and returned to a normal family, social and professional life within the 3 months following reoperation, with no epilepsy The pathological examination confirmed a WHO grade II oligodendroglioma, with no sign of malignant transformation. No oncological medical treatment was given. Currently, with 12 years of follow-up, the patient is still asymptomatic, active, with a stable MRI.

Firstly, in patients who underwent awake connectome-based surgery, survival was significantly improved in diffuse high-grade and low-grade gliomas (26), especially with the first ever-published report of a median OS>20 years (not yet reached) for LGG (6). Here, “connectome” means that the actual functional distribution of cerebral circuits was mapped in real-time in patients who achieved a multi-tasking with time constraint throughout resection, based on a continuous cognitive examination reflecting the actual interplay between functional systems (27). Secondly, by postponing postoperative adjuvant treatment, especially RT (28), it was possible to reoperate patients (from one to 4 further surgeries over years) (29), and to increase each time the EOR without inducing neurological or neurocognitive deterioration (30), thanks to mechanisms of circuits redeployment that occurred over time, as evidenced by remapping from operation to operation (31) (**Figure 1**). Such a multistep strategy enabled a significant improvement of OS, both in LGG and high-grade gliomas, with a median OS close 18 years after 3 consecutive surgeries in grade 2–3 glioma patients (32). Thirdly, QoL was maintained, not only by limiting the risk of severe permanent deficit <1%, but also by preserving cognition, as evidenced by objective neuropsychological assessments performed after awake glioma resection, following functional rehabilitation (33). Long-term activities were also preserved, with a KPS score ≥80 during at least 14–15 years in LGG patients (6, 7, 34). Familial stability was maintained in most patients, with a rate of divorce <8% after surgery (35). About 93-94% of patients were able to resume their employment following awake surgery, regardless the socio-professional categories (6, 34). Interestingly, patients who returned to work postoperatively were more likely to undergo reoperation(s) and had a longer OS (36). These original data about 515 glioma patients suggest that a better QoL is correlated with a prolonged survival, showing that the optimal onco-functional balance can be found by means of “functional connectome-guided surgery”, solving the traditional dilemma between QoL/OS which are in fact not antagonist but linked (36). Intraoperative monitoring of higher cognitive and emotional functions allowed students to be graduated in 95% of cases (37), sportspeople to resume (intensive) sport activities in >92% of cases (38), or even to remain creative, by practicing various arts after surgery, e.g., music, painting, architecture, dance… (39). Functional-mapping based surgery also resulted in the elaboration of new models of cognitive neurosciences, by proposing a meta-networking theory of brain processing, paving the way of an improved knowledge of pathophysiological mechanisms underpinning neuroplastic potential (24, 40). Data gained from intraoperative stimulation mapping were combined with longitudinal functional neuroimaging findings collected before and after resection (41) – since fMRI and DTI represent excellent tools for fundamental research outside the OR (42). Based on these multimodal studies, original functional atlases of cortico-subcortical networks critical for human brain functions were developed, emphasizing that white matter tracts represent the main limitation of neuroplasticity (43, 44).

In conclusion, the future of neurooncology lies on basic neurosciences rather than on surgical technologies, that should be used in a research perspective rather than to compensate a lack of knowledge about functional anatomy – with a risk for young neurosurgeons to become dependent on the machine. To avoid such an addiction, and to learn how to use technology appropriately, students in neurosurgery who would like to be specialized in brain pathologies must be educated very early in neurosciences. By developing a hybrid training program allowing them to speak both languages of surgery and fundamental sciences since the beginning and to the end of their carrier, they could evolve toward a new field of “functional connectome-based neurooncology” (45).
